# Gender-specific temporal trends in overweight prevalence among Chinese adults: a hierarchical age-period-cohort analysis from 2008 to 2015

**DOI:** 10.1186/s41256-020-00169-w

**Published:** 2020-09-11

**Authors:** Yinmei Yang, Mohammedhamid Osman Kelifa, Bin Yu, Carly Herbert, Yongbo Wang, Junfeng Jiang

**Affiliations:** 1 School of Health Sciences, Wuhan University, 115 Donghu Road, Wuhan, 430071 Hubei China; 2 Department of Epidemiology, University of Florida, 2004 Mowry Road, Gainesville, FL 100231 USA; 3 University of Massachusetts Medical School, Worcester, MA USA

**Keywords:** Overweight, Chinese adults, Gender difference, Age-period-cohort model

## Abstract

**Background:**

As a key health risk, the prevalence of overweight has been strikingly increasing worldwide. This study aimed to disentangle the net age, period, and cohort effects on overweight among Chinese adults by gender.

**Methods:**

Data came from the Chinese General Social Survey from 2008 to 2015, which was a repeated cross-sectional survey (*n* = 55,726, aged 18 and older). χ^2^ or *t* tests were used to estimate the gender disparities in overweight and socioeconomic status (SES). A series of hierarchical age-period-cohort cross-classified random-effects models were performed using SAS version 9.4 to estimate the overall and gender-specific temporal trends of overweight, as well as the association between SES and overweight. Further, a series of line charts were used to present the age and cohort variations in overweight.

**Results:**

After controlling for covariates, significant age and cohort effects were observed among adults in China (b = 0.0205, *p* < 0.001; b = 0.0122, *p* < 0.05; respectively). Specifically, inverted U-shaped age effects were identified for both genders, with a high probability of overweight occurring in middle age (b = –0.0012, *p* < 0.001). Overweight was more prevalent among men than women before 60 years old, and this trend reversed thereafter (b = –0.0253, *p* < 0.001). Moreover, men born during the war (before 1950) and reform cohorts (after the 1975s) demonstrated a substantial decline in overweight, while men born in 1950–1975 showed an increasing trend in overweight prevalence (b = 0.0378, *p* < 0.05). However, the cohort effect on women was not statistically significant. Additionally, a higher SES was related to an elevated probability of overweight.

**Conclusion:**

Gender-specific age and cohort effects on the prevalence of overweight were observed among Chinese adults. Both China and other developing countries need to pay attention to the coming obesity challenge and related health inequality. Full life-cycle overweight prevention interventions should focus on middle-aged adults, men born in the war and reform eras, and adults with a higher SES.

## Introduction

Overweight is a public health challenge worldwide [1]. Despite great progress in the prevention and control of obesity and overweight [2], overweight prevalence among male and female adults in China has reached as high as 30.1 and 34.5%, respectively [3]. Overweight is positively associated with various chronic diseases, including diabetes, hypertension and cancer [4–6], and contributes to a growing burden and loss of health-related quality of life, premature mortality, and healthcare expenditures [7–9]. However, the causes of overweight are complex. Environmental, behavioral, and socioeconomic status (SES) factors have been postulated to play dominant roles [2, 10–13].

Numerous studies have explored the effects of age, period, and cohort on overweight prevalence separately or in pairs [14–17]. However, limited research has simultaneously examined these effects on overweight prevalence, especially cohort effect, due to the exact linear dependency among age, period, and cohort. The hierarchical age-period-cohort-cross-classified random-effects model (HAPC-CCREM) offers a potential approach to account for the contributions of age, period and birth cohort, and to adjust for confounding factors [18, 19]. Several studies to date have focused on topics regarding body mass index (BMI) using the APC model. For instance, significant age, period and cohort effects on overweight were observed in Australian adults from 1990 to 2000 [20]. Using the China Health and Nutrition Survey data from 1989 to 2009, Fu and Land [21] found an inverted U-shaped relationship between age and overweight prevalence, indicating a peak rate of overweight in middle age. Additionally, Robinson et al. [22] noted that the baby boomers in the United States showed a higher cohort-specific risk for abdominal obesity. Both literature from Australia and China identified that more recent generations or cohorts were at heightened risk for overweight and obesity [20, 23]. These studies highlight the significance of interventions in early life to curb the adulthood overweight epidemic. Despite important findings from extant research, the temporal trend in overweight prevalence among Chinese adults remains unclear, especially in recent years.

A literature review has also reported that overweight varies by gender [24]. In general, the prevalence of overweight was more common among men than women in China [3, 15]. Furthermore, relatively little is known about the gender-specific temporal trends in overweight. For example, in China, men experience a more rapid tempo of population overweight prevalence than women [21]. However, similar age and period effects on overweight prevalence were identified for both genders in Australia, but the contribution of birth cohort on overweight was significant only in women [20].

After a comprehensive review, it seems few studies have examined the cohort effect on overweight with age and period controlled for, and no study has discussed the gender disparities in overweight across successive cohorts in China. Accordingly, based on nationwide data from the Chinese General Social Survey (CGSS), the current study applied HAPC-CCREM to examine temporal trends in the prevalence of overweight among Chinese adults, as well as their gender disparities.

## Methods

Data were collected from the CGSS, which was a repeated cross-sectional survey in China. A detailed procedure about CGSS has been described previously [25, 26]. Briefly, this survey has been conducted since 2003, using a multi-stage stratified probability proportionate to size sampling method to investigate Chinese adults (age ≥ 18). This study pooled data from the years 2008 (*n* = 6000), 2010 (*n* = 11,783), 2011 (*n* = 5620), 2012 (n = 11,765), 2013 (*n* = 10,724), and 2015 (n = 10,968). Height and weight were not available for CGSS2003, CGSS2005, and CGSS2006; hence, these surveys were excluded. Missing data for 1134 cases were deleted. The final sample included 55,726 adults.

Self-rated height and weight were recoded into meter and kilogram in all surveys. BMI was calculated as weight divided by height squared (kg/m^2^). The dependent variable was overweight using the cut-off point (BMI ≥ 24 kg/m^2^) recommended by the Working Group on Obesity in China [27]. The key independent variables included age, age square, period, and cohort. Age was treated as a continuous variable. Cohort was divided into 5-year intervals, and 14 birth cohort groups were yielded from pre-1929 to 1990-. Period contained six waves (2008, 2010, 2011, 2012, 2013, and 2015).

This study also collected data on gender (men or women), ethnicity (Han or minority), education (low education or high education), household income (very low, low, medium, high, or very high), and residence (urban or rural). High education was defined as the completion of college and all degrees beyond that point. In China, urban dwellers usually have better access to opportunities, economic and social benefits. Therefore, living in urban settings, high education, and high or very high household income were considered as proxies for a higher SES in the present study.

The HAPC-CCREM was used to address the identification problem due to the exact collinearity (cohort = period–age). This method was firstly used by Yang and Land [19] for repeated cross-sectional survey data in 2006. In HAPC-CCREM, period and cohort are treated as level 2 variables, while age is considered as level 1 variable. The multilevel design of this method can effectively disentangle the identification problem among age, period, and cohort, because variables at different levels are not directly additive [18]. In the current study, a series of models were constructed to estimate the net effects of age, period, and cohort. Age, residence, ethnicity, education, and household income were considered as fixed effects (level-1), whereas period, cohort and gender were included as random effects (level-2). According to prior research, overweight was assumed to follow a quadratic function of age [28]. Gender, age, residence, education, and household income were centered around their grand means to facilitate the interpretation and reduce collinearity problems between main effects and their interactions. In addition, gender differences were examined using t-test or Chi-square test. Analyses were weighted to account for sample design. All statistical analyses were performed using SAS version 9.4.

## Results

Relative to women, men were more likely to be older, overweight, minority, have high education, live in rural areas and have more household income. Details are listed in Table 1. Period-cohort-specific overweight prevalence rates among Chinese adults are presented in Table 2. Pronounced gender differences in overweight were found, with more fluctuations among men. Additionally, the overweight prevalence was positively related to age (*p* <  0.001) and inversely associated with age^2^ (*p* <  0.001), suggesting an inverted U-shaped relationship between age and overweight. The random effects of residual variance components suggested a significant cohort effect but a non-significant period effect of overweight. Similar results were found in the full model after controlling for all covariates.

**Table 1:** Descriptive statistics of variables used, n (%)

Variables	Overall	Men	Women	*p* value
**Total**	55,726 (100.0)	27,344 (49.1)	28,382 (50.9)	
**Age (years), Mean (SD)**	48.0 (16.1)	48.6 (16.2)	47.4 (16.0)	< 0.001
**Overweight**				< 0.001
No	38,624 (69.3)	18,220 (66.6)	20,404 (71.9)	
Yes	17,102 (30.7)	9124 (33.4)	7978 (28.1)	
**Residence**				< 0.001
Rural	26,621 (47.8)	13,277 (48.6)	13,344 (47.0)	
Urban	29,105 (52.2)	14,067 (51.4)	15,038 (53.0)	
**Ethnicity**				< 0.001
Han	51,269 (92.0)	26,056 (91.8)	25,213 (92.2)	
Minority	4457 (8.0)	2326 (8.2)	2131 (7.8)	
**Education**				0.001
Less than college	46,964 (84.3)	22,617 (82.7)	24,347 (85.8)	
College or above	8762 (15.7)	4727 (17.3)	4035 (14.2)	
**Household income**				< 0.001
Very low	3748 (6.7)	1862(6.8)	1886 (6.6)	
Low	18,080 (32.4)	8784 (32.1)	9296 (32.8)	
Medium	29,527 (53.0)	14,355 (52.5)	15,172 (53.5)	
High	4187 (7.5)	2252 (8.2)	1935 (6.8)	
Very high	184 (0.3)	91 (0.3)	93 (0.3)	

Note: *SD* standard deviation

**Table 2:** Overweight prevalence by period and cohort among adults in China, CGSS2008–2015

Cohort	Period											
	2008		2010		2011		2012		2013		2015	
	n	%	n	%	n	%	n	%	n	%	n	%
Men												
Pre-1929	12	41.7	92	20.7	45	22.2	82	14.6	52	13.5	47	17.0
1930-	36	19.4	174	25.9	61	26.2	151	23.2	135	27.4	123	28.5
1935-	63	42.9	246	28.0	108	19.4	276	24.6	217	18.4	178	20.8
1940-	178	32.6	314	27.7	154	28.6	377	26.8	280	25.4	273	28.9
1945-	224	34.4	444	30.0	197	32.5	483	29.0	407	28.3	408	32.6
1950-	286	34.3	564	30.9	260	36.5	647	32.5	554	33.4	520	35.6
1955-	270	29.3	582	36.4	277	36.1	548	34.9	506	35.0	501	32.3
1960-	340	33.8	609	38.8	271	40.2	670	38.8	559	38.1	482	36.9
1965-	360	39.7	635	41.6	246	36.6	652	37.7	533	34.0	528	43.8
1970-	348	36.5	653	38.9	278	43.9	644	40.4	587	41.1	471	43.3
1975-	257	35.8	415	41.0	188	42.6	429	38.0	430	36.7	381	41.2
1980-	272	20.2	399	32.8	170	41.8	403	35.7	368	35.6	316	44.0
1985-	201	13.9	333	23.7	173	24.9	349	28.9	375	28.3	349	30.4
1990-	28	17.9	159	10.7	90	15.6	249	14.9	332	16.9	460	21.5
Total	2875	31.9	5619	33.6	2518	34.9	5960	33.0	5335	32.2	5037	34.8
Women												
Pre-1929	15	26.7	74	28.4	43	14.0	83	22.9	56	19.6	60	13.3
1930-	22	31.8	157	31.8	70	28.6	151	18.5	105	21.9	123	35.0
1935-	52	50.0	213	31.9	124	29.8	202	26.2	191	27.7	205	31.7
1940-	150	44.7	286	31.5	153	34.6	277	33.6	297	34.7	276	31.2
1945-	223	39.9	432	35.2	218	35.8	380	32.9	312	34.6	432	35.0
1950-	276	42.8	557	35.5	272	35.7	553	40.0	514	37.0	541	39.2
1955-	314	33.1	583	35.3	297	40.1	515	40.8	476	39.1	516	36.8
1960-	338	33.4	690	36.1	318	35.2	549	36.4	514	34.6	533	37.3
1965-	399	27.6	732	31.0	329	29.5	618	32.7	535	34.2	701	31.0
1970-	438	21.9	714	26.1	370	27.6	652	24.8	570	27.4	556	32.6
1975-	301	16.9	528	19.9	225	20.9	476	21.2	471	25.9	446	21.1
1980-	278	10.8	443	13.8	222	20.3	470	20.4	463	17.3	366	18.6
1985-	242	5.4	400	7.3	205	11.7	383	12.0	358	12.0	409	18.6
1990-	31	3.2	162	4.3	107	1.9	287	5.6	301	7.3	456	9.0
Total	3079	26.9	5971	27.6	2953	28.4	5596	28.1	5163	28.2	5620	29.0

**Fig. 1:**
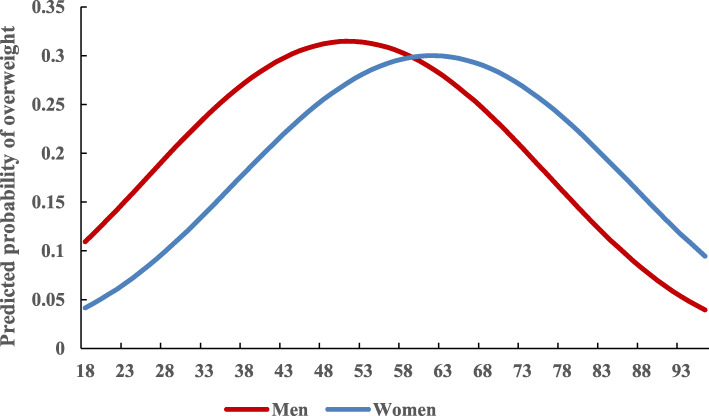
Gender-specific age effects on overweight, adjusted for period and cohort effects and all covariates

Inverted U-shaped relationships were observed between overweight and age for both men and women (Fig. 1). Specifically, the overweight rate tended to increase until middle age, and decrease thereafter. Compared to women, the overweight prevalence was significantly higher for men, but this pattern reversed after 60 years old.

**Fig. 2:**
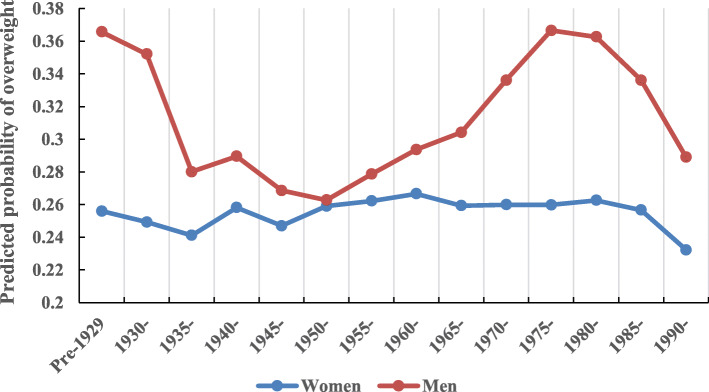
Gender-specific cohort effects on overweight, adjusted for age and period effects and all covariates

As shown in Fig. 2, changes in overweight rates were not uniform, with substantial differences by gender across successive cohorts. Overall, the magnitude of cohort effect appeared to be more pronounced among men. Specifically, the prevalence of overweight among men demonstrated a sharp decrease in cohorts before 1950, and then an increasing overweight trend in generations born in 1950–1975. This was followed by a rapid decline in overweight prevalence among cohorts after 1975. Despite some small fluctuations, the overweight prevalence for women remained relatively stable across successive cohorts.

**Fig. 3:**
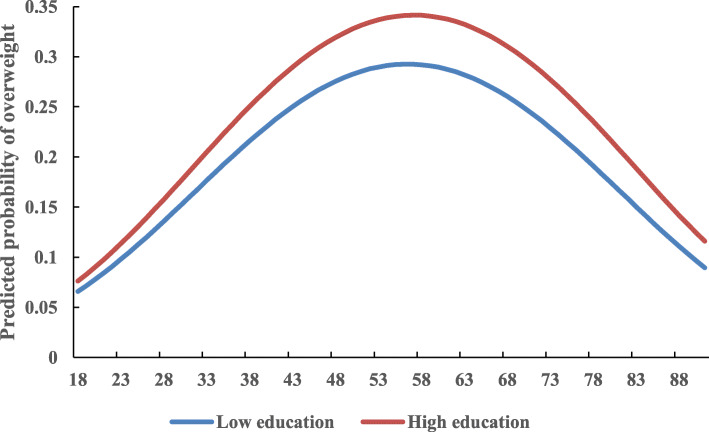
Education-specific age effects on overweight, adjusted for period and cohort effects and all covariates

Individuals with high education had a consistently higher prevalence of overweight than their counterparts with low education (Fig. 3). Moreover, individuals living in an urban setting and a household with higher income were more likely to be overweight (Table 3, model 5). Therefore, individuals with a higher SES were at an increased risk for overweight.

**Table 3:** Estimates from HAPC-CCREM of age, period and cohort effects on overweight among Chinese adults, CGSS2008–2015

	Model 1	Model 2	Model 3	Model 4	Model 5
**Fixed effects**					
Intercept	−0.5697^***^ (0.0507)	−0.9583^***^ (0.0581)	−0.9282^***^ (0.0592)	−0.9678^***^ (0.0583)	−0.9344^***^ (0.0580)
Age	0.0166^***^ (0.0017)	0.0181^***^ (0.0017)	0.0206^***^ (0.0018)	0.0207^***^ (0.0018)	0.0205^***^ (0.0017)
Age^2^	−0.0012^***^ (0.0001)	− 0.0012^***^ (0.0001)	− 0.0013^***^ (0.0001)	−0.0013^***^ (0.0001)	− 0.0012^***^ (0.0001)
Ethnicity (ref. Han)		−0.1486^***^ (0.0359)	−0.1474^***^ (0.0361)	− 0.1423^***^ (0.0357)	− 0.1482^***^ (0.0361)
Education (ref. less than college)		0.1348^***^ (0.0289)	0.2155^***^ (0.0307)	0.1387^***^ (0.0292)	0.2100^***^ (0.0306)
Residence (ref. rural)		0.1210^***^ (0.0191)	0.1234^***^ (0.0196)	0.1234^***^ (0.0192)	0.1226^***^ (0.0196)
Gender (ref. women)		0.1973^***^ (0.0187)	0.2357^***^ (0.0200)	0.2493 (0.1540)	0.2824^**^ (0.0583)
Household income		0.1486^***^ (0.0129)	0.1486^***^ (0.0130)	0.1518^***^ (0.0130)	0.1480^***^ (0.0130)
Age^*^gender			−0.0306^***^ (0.0013)		−0.0253^***^ (0.0028)
Age^*^residence			0.0004 (0.0013)		0.0003 (0.0013)
Age*education			0.0186^***^ (0.0020)		0.0180^***^ (0.0020)
Age^*^gender^*^residence			0.0001 (0.0025)		−0.0001 (0.0025)
Age^*^gender^*^education			−0.0266^***^ (0.0038)		−0.0242^***^ (0.0039)
**Random effects variance components**					
**Period effect**					
Intercept	0.0048 (0.0035)	0.0029 (0.0023)	0.0025 (0.0021)	0.0020 (0.0017)	0.0026 (0.0021)
**Cohort effect**					
Intercept	0.0123^*^ (0.0067)	0.0122^*^ (0.0065)	0.0136^*^ (0.0074)	0.0136^*^ (0.0071)	0.0122^*^ (0.0069)
Gender				0.3236^**^ (0.1306)	0.0378^*^ (0.0199)

***p < 0.001, **p < 0.01, *p < 0.05

## Discussion

Significant age and cohort effects on overweight prevalence were identified among Chinese adults. In line with prior research [29, 30], overweight appears to be more prevalent among middle-aged adults. Physiological changes, including hormonal changes, decreased metabolism and reduced physical activity, may account for the higher risk of overweight in this age group [31, 32]. There was a lower prevalence of overweight among older adults, which may be explained by decreased food intake, reduced hunger sensations, and loss of appetite that accompanies aging [33]. The present study also found that overweight rates were substantially higher among men compared to women in early and middle adulthood, and this trend reversed after 60 years old, with more women being overweight than men in late adulthood. The gender difference can be ascribed to both biological and social factors. Younger women are more susceptible to social pressures of body shapes, touting slimness as a sign of feminine beauty [34], whereas men have more social acceptance of larger body sizes [35]. On the other hand, the menopausal transition affects the distribution of body fat, and menopause is associated with a tendency to weight gain [36, 37]. Given that the average age of menopause is about 50 years in China [38], this is likely to play a role in our findings.

This study found no evidence in support of period effect on overweight prevalence, with rates of overweight among Chinese adults leveling off at 30%. This is inconsistent with a Chinese study that indicated an increasing trend of overweight from 1989 to 2009 [21]. This stabilization may be an early sign of a plateau in the overweight epidemic among Chinese adults in more recent years. It is possible that the overweight prevalence in more recent periods varies less, similar to research in developed countries and a study conducted in Guangdong, China between 2002 and 2010 [39, 40]. Alternatively, a 7-year period may not be long enough to capture a significant period effect of overweight prevalence.

Rapid economic and social transitions have dramatic effects on Chinese men born in different years, and these factors may help explain the significant cohort effects of overweight identified in this study. Birth year is likely to impact the landscape of one’s early childhood development, which is one proposed mechanism of the differential prevalence of overweight among cohorts. Overweight prevalence showed a sharp decline among men born before 1950, a period of economic and social instability due to wars, which may have negatively impacted their fetus period, childhood, and adolescence. Individuals growing up amongst chronic nutritional deprivation due to food scarcities have a predisposition to accumulating fat mass in later life, which may provide some explanations to this finding. The developmental origins hypothesis also proposes that malnutrition during these critical periods can exert permanent impacts on later adult health, including obesity [41]. Recently, a study revealed that fetal and early childhood exposure to the Korean War significantly predicted increased BMI in adulthood, due to the mismatch between the early and later life nutritional status and availability [42]. Moreover, the greater the mismatch between early and later life nutritional status, the greater the risk of increased BMI [43]. People born during or just before the wars were more vulnerable to experience malnutrition than those born during stable eras [42]. Hence, men born in China during the first half of the twentieth century experienced greater early childhood malnutrition, increasing their risk of overweight later in life due to mismatch. This mismatch decreased gradually for later cohorts. Also, according to “survival bias”, people born before 1950 now belong to the elderly group; hence, only healthier individuals could survive and participate in surveys.

Our findings also suggested that men born during 1970-1975 showed a dramatic upward trend in overweight prevalence, which may be attributed to substantial lifestyle changes due to technological and environmental advancement, such as increasing sedentary lifestyles and less physically demanding work [20, 31, 44], as well as more food to meet people’s basic nutrition needs in the planned economy. Since the implementation of the Reform and Opening-up policy in 1978, China has been one of the fastest growing economies in the world. Our results revealed that the overweight prevalence among Chinese adults decreased rapidly in the most recent birth cohorts (after 1975). One study in Taiwan, China showed that individuals born after 1970 were more likely to report a lower BMI compared with their older counterparts (born before 1970), due to some counteractive impacts of economic development [34]. This is possibly explained by the most recent cohorts’ ability to possess more health capital to generate improved physiological capacity, such as greater access to health information, more attention to body shape and awareness of harmful effects of overweight on health [23].

Moreover, the cohort effect demonstrated marked changes in males, with fewer variations in females. The gender difference may be explained by son preference. The root of son preference lies deep in Chinese traditional culture [45]. The developmental origins hypothesis states that the malnutrition in utero may predispose to overweight in later years [41]. The thrifty phenotype hypothesis also proposes that an undernourished baby usually becomes thrifty to adapt to an adverse environment [46]. Additionally, some researchers noted that these adaptations only became detrimental when nutrition was more abundant in the postnatal environment than the prenatal environment [47, 48]. Before the Reform and Opening-up, most families in China were in a state of lack of material, and high fertility. In this case, girls were more susceptible to malnutrition relative to boys in settings of family food shortages, owing to the son preference. In that particular historical, cultural and developmental contexts, male nutrition was superior to their female counterparts in the postnatal environment. The mismatch between early and later life nutritional status results in an increased risk of overweight in male adulthood. This situation was alleviated with the improvement of material conditions after the Reform and Opening-up.

Our findings also demonstrated that a higher SES was positively associated with overweight among Chinese adults, which is supported by a cross-sectional study in Kenya [49]. This phenomenon may be explained by the availability of excess food and less physically demanding work for high-SES groups [50].

Several limitations of our study should be mentioned. First, height and weight in the CGSS were self-reported; hence, overweight may be underestimated due to social desirability bias. Second, given the relatively short period of data collection, caution should be exercised in interpreting these results. A longer period may help capture the period effect on overweight. Furthermore, causal relationships cannot be elucidated by the usage of APC analysis of the repeated cross-sectional study. Finally, other variables like physical exercise and living environments are not available for all surveys; hence, we only investigated the impacts of demographic and SES characteristics on overweight.

Despite the above limitations, our findings have several significant implications to help guide future programs and policies in China. Firstly, targeted courses and trainings should be tailored to specific subpopulations (e.g., by SES and gender), to address the overweight epidemic and narrow health disparities. Secondly, as middle-aged adults, men in war and reform cohorts are vulnerable to overweight, improving obesogenic environments for them should be prioritized. Finally, given the overweight epidemic among Chinese adults, large-scale, health promotion activities are urgently needed [51].

## Conclusions

In conclusion, the results reveal that overweight prevalence remains stable in recent years, whereas significant gender-specific age and cohort effects were observed among Chinese adults. The cohort effect was particularly pronounced among Chinese males, reflecting the indelible impact of early life experiences on Chinese men’s overweight. In addition, there was a significant relationship between higher SES and overweight. Overweight or obesity has been increasingly prevalent in developing countries over the past several decades. The overweight transition among Chinese people can provide some useful lessons and insights to other developing countries. Therefore, full life-cycle overweight interventions can warrant the immediate attention of policy makers in not only China but also other similar contexts, and particular attention should be paid to middle-aged adults, men in war and reform cohorts, and adults with a higher SES.

## Data Availability

The data used during the current study are available from the corresponding author on reasonable request.
